# The Evolution and the Advantages of MicroED

**DOI:** 10.3389/fmolb.2018.00114

**Published:** 2018-12-12

**Authors:** Brent L. Nannenga, Guanhong Bu, Dan Shi

**Affiliations:** ^1^School for Engineering of Matter, Transport and Energy, Arizona State University, Tempe, AZ, United States; ^2^Structural Biophysics Laboratory, National Frederick Laboratory for Cancer Research, National Cancer Institute, Frederick, MD, United States

**Keywords:** Cryo-EM, crystallography, MicroED, dynamic scattering, radiation damage

## Abstract

MicroED is a method which combines cryo-EM sample preparation and instrumentation, with electron and X-ray crystallography data analysis, and it has been employed to solve many protein crystal structures at high resolution. Initially, the main doubts of this method for structure determination were the dynamic scattering of electrons, which would cause severe inaccuracies in the measured intensities. In this paper, we will review the evolution of MicroED data collection and processing, the major differences of multiple scattering effects in protein crystals and inorganic material, and the advantages of continuous rotation data collection. Additionally, because of the periodic nature of the crystalline sample, radiation doses can be kept significantly lower than those used in single particle data collection. We review the work where this was used to assess the radiation damage of a high-energy electron beam on the protein molecules at much lower dose ranges compared to imaging.

## Introduction

Cryo-EM has become one of the most powerful tools in structural biology after nearly four decades of improvements in electron optics, direct electron detectors, and software (Frank, 2016). The major break-through technologies that have enabled the wide-spread adoption of cryo-EM are the computerized and newly designed electron optic systems and the introduction of direct electron detectors (Ruskin et al., 2013; Cheng et al., 2015). These technologies have facilitated the automated collection of large single particle data sets, and the development of the new software to reduce the effects caused by specimen motion (Grigorieff, 2013; Li et al., 2013). With these advances, large structures can routinely be determined at resolutions that allow the modeling of amino acid side chains. In addition to single particle cryo-EM, electron crystallography has been used to determine 2D membrane crystal structure at high resolution (Gonen et al., 2005). 3D crystals were also analyzed by electron crystallography, however for many years these samples resisted structure determination (Dorset and Parsons, 1975; Unwin and Henderson, 1975; Shi et al., 1998; Jiang et al., 2009). In 2013, micro electron diffraction, or MicroED, was developed and used to determine the first structure of a protein from a thin 3D microcrystal (Shi et al., 2013). The MicroED technique is used to collect high-resolution electron diffraction movie data sets from sub-micrometer sized 3D protein crystals at extremely low-dose (Nannenga and Gonen, 2016). The new advantage of MicroED is that the electron diffraction movies collected using the continuous rotation method (Nannenga et al., 2014b) can then be processed by standard X-ray crystallographic programs. In recent years, electron diffraction methods become a valuable tool and has been used to determine biomolecular structures, in some cases at sub-angstrom resolution(Nannenga et al., 2014a; Rodriguez et al., 2015; Yonekura et al., 2015; Sawaya et al., 2016; Krotee et al., 2017; Gallagher-Jones et al., 2018; Guenther et al., 2018; Hughes et al., 2018; Liu and Gonen, 2018; Seidler et al., 2018; Xu et al., 2018), and applied to material science to novel structures (Mugnaioli et al., 2009; Simancas et al., 2016; Palatinus et al., 2017; Vergara et al., 2017; Yuan et al., 2018; Zhang et al., 2018). The general applicability of electron diffraction techniques to all these samples has been made possible by continued method development and optimization. The evolution of MicroED methods are briefly described in the following section.

## The Evolution of Data Collection and Processing

In the first proof of concept MicroED study (Shi et al., 2013), still diffraction patterns were collected at discrete angles from multiple lysozyme crystals, and the data was processed and merged manually. Due to the nature of still diffraction patterns, most of the recorded intensities were only partially sampled. To collect full intensities, the continuous rotation (CR) method for MicroED was developed, in which the compustage of the cryo-TEM is continuously rotated at a constant speed. Initially, the tilting speed of the microscope was controlled by the force applied to the F20 (FEI/ThermoFisher) alpha tilt buttons. After few tests, a constant speed of 0.09 degrees per second was generated by a weight, as shown in the Supplementary Video. The rotation rate can be seen by the alpha value in the lower-right corner of the screen. This approach was the first used for the MicroED CR method, and it was applied to the same lysozyme crystals used in the first study (Nannenga et al., 2014b), as well as the study of catalase and α-synuclein peptide fragments (Nannenga et al., 2014a; Rodriguez et al., 2015). The next iteration of CR rotated the stage using a home designed device (Shi et al., 2016), and this was followed by the third generation of CR where the rotation is controlled via software embedded in ThermoFisher/FEI microscopes. Other related electron diffraction techniques have also made use of continuous rotation data collection to improve the resulting data quality (Nederlof et al., 2013; Gemmi et al., 2015). In the initial MicroED study, a combination of manual indexing and in-house developed programs based on previous algorithms (Shi et al., 1998) for integration were used to generate merged intensity of still diffraction data (Iadanza and Gonen, 2014). Since CR was developed, all diffraction movies from the rotating protein crystals can be easily processed through previously developed X-ray crystallographic software [e.g., Mosflm (Hattne et al., 2015), XDS (Rodriguez et al., 2015)], and DIALS (Clabbers et al., 2018). Detailed protocols on sample preparation, data collection, and processing have been published previously (Hattne et al., 2015).

## Multiple Scattering and Dynamic Scattering in Protein Crystals and Inorganic Crystals

**Figure 1:**
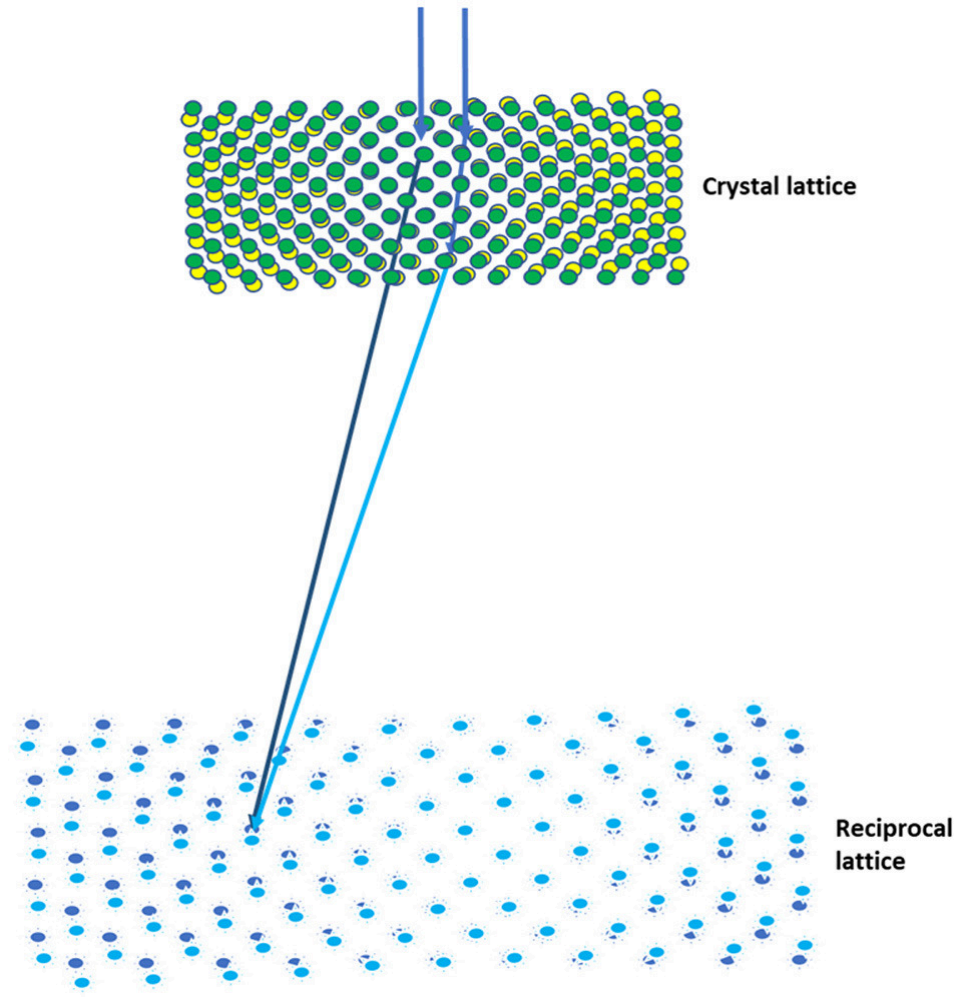
Continuous rotation data collection in MicroED. The effects of multiple scattering (light blue) can be reduced using the CR method, as both the crystal and the reciprocal lattices are being rotated while the scattered electrons traveling in the crystal.

Because of the strong interaction between electrons and the sample, the dynamical scattering of electrons in a crystal has been a major hurdle in the recording of accurate intensities (Spence, 1993). To overcome this, the precession electron diffraction (PED) technique was developed for diffraction data collection (Vincent and Midgley, 1994), and this has been shown to dramatically decrease the effects of dynamic scattering relative to traditional electron diffraction patterns (Oleynikov et al., 2007). The rotation electron diffraction (RED) method was developed to determine the structures of inorganic crystals using fine step rotation and small-angle beam tilting (Wan et al., 2013), in which the diffraction data was off-zone axis patterns and might contain less overall dynamic scattering events. Similarly, the CR method developed for MicroED was shown to reduce the effects of dynamic scattering included on the zone axes of reciprocal space and yield more accurate structures (Nannenga et al., 2014b). Because the crystal is rotating as the data is being collected, the allowed secondary scattering events are reduced as the Bragg reflections are being integrated. Also, both the PED and the CR methods employ the relative movement between the Ewald sphere and the reciprocal lattice to scan reciprocal space, facilitating the collection of full diffraction intensities. The subtle difference between these two methods is that the crystal and its reciprocal spaces are continuously rotating while the scattered electron traveling in the crystal for the CR, as shown in Figure 1, and both the real and its reciprocal spaces are static to the scattered electrons in the PED method. In other words, the continuously rotation could generate an additional subtle tilting of the crystal to the dynamically scattered electrons. It would be interesting to compare the electron diffraction data from the same crystals using the PED and the CR methods on the major zone axis.

In addition to reducing dynamic scattering through data collection strategies, the difference in atomic composition of biological crystals (light atoms) relative to inorganic crystals (heavier atoms) also plays an important role in reducing the dynamic scattering. When thin catalase crystals (~200 nm thick) were used to assess the scattering observed for MicroED data, it was found that the ratio of elastically scattered electrons relative to un-scattered electrons (direct beam) was low (0.2), indicating that the kinematic assumption is appropriate. The results of this analysis were very similar to those obtained in previous work on catalase (Dorset and Parsons, 1975; Unwin and Henderson, 1975). These also agree on the fact that the elastic scattering mean free path of the high energy electrons in the cryo-biological samples are at least 4 times longer than in the inorganic materials with atomic number bigger than 25 (Grimm et al., 1996; Iakoubovskii and Mitsuishi, 2009), which means that the multiple scattering events could be less frequent in the protein crystals than in the inorganic crystals. Together, this could explain why biological samples with CR method do not appear to suffer from dynamic scattering as much as is seen in inorganic crystals (Spence, 1993).

## Using MicroED to Assess Radiation Damage

The radiation damage caused by high-energy electrons when they interact with beam sensitive material is the key reason for the resolution limitations in cryo-EM. Both protein crystals (Stark et al., 1996; Baker et al., 2010) and single particles (Bartesaghi et al., 2014; Grant and Grigorieff, 2015) have been used to estimate radiation damage. The radiation damages are more likely accumulated from high energy electron beam knocking out the electrons of protein molecules in vitreous ice, it would be important to assess the damage starting from lower dose. The signal of a diffraction experiment increases with the square of the number of unit cells in the crystal; therefore, MicroED can obtain accurate 3D density maps at high resolution using total doses of as low as ~1 e/Å^2^, which allows the analysis of radiation damage effects in both reciprocal space and real space (Hattne et al., 2018).

**Figure 2:**
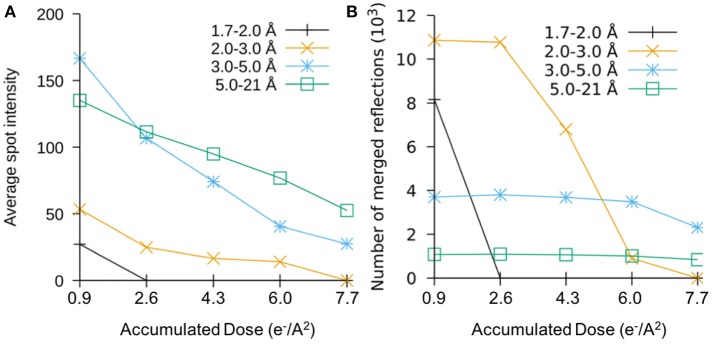
The plots of sweep number vs. **(A)** intensity of average per spot and **(B)** total number of measurable reflection numbers in the different resolution sphere shells of merged 3D reciprocal lattice, each resolution bin was colored differently.

A study on the effects of radiation damage at these very low doses was conducted by collecting data over the same rotation range from the same crystals in increments of 1.6 e/Å^2^ total dose (Hattne et al., 2018). Figures 2A,B show the plots of the averaged intensities and the number of merged reflections in different resolution bins as the dose accumulates. The number of reflections rapidly decreases in the high-resolution shells as the fine features of the crystals lattice are lost to radiation damage, and the total intensities gradually decay at resolution-dependent rates as the dose accumulates. After a very small amount of dose has accumulated, there are no measurable reflections beyond 2 Å, which indicated the most of high-resolution information has already been lost. When the accumulated dose reaches > ~8 electron/Å^2^, most information at better than 3 Å in resolution is also lost. In this study, the effects of site-specific radiation damage were also found to follow a similar trend indicating that these effects are not solely do to a loss in crystalline order. This suggests that radiation damage at the atomic scale could occur at much higher rates than what has been estimated using single-particle measurements (Bartesaghi et al., 2014; Grant and Grigorieff, 2015).

## Discussion

Since the initial presentation of MicroED, the data collection, and processing methods have evolved to continually improve the method. The use of MicroED has several advantages for structure determination including the ability to determine structures from a small number of crystals (in some cases a single crystal (Nannenga et al., 2014b) that are several orders of magnitude smaller than those needed by traditional crystallographic methods. A unique advantage of electron diffraction is that it is very sensitive to charge and chemical bonding (Chang et al., 1999; Wu and Spence, 2003; Yonekura and Maki-Yonekura, 2016). When these effects are properly accounted for and modeled, MicroED could be used to directly visualize charge and bonding in protein structures. Specimen preparation usually is the bottle neck for MicroED because of the fragileness of protein crystals, using cryo-FIB/SEM to reshape large proteins crystals embedded in ice without blotting is a very promising method (Duyvesteyn et al., 2018), few other groups are also working on the similar approach. Continued development of the method promises to cement electron diffraction's status as a unique and valuable tool for structural biology and materials characterization.

## Author Contributions

DS, BN, and GB contributed to the writing of this paper.

### Conflict of Interest Statement

The authors declare that the research was conducted in the absence of any commercial or financial relationships that could be construed as a potential conflict of interest.

## Supplementary Material

Supplementary Video The first generation of continuous rotation device - from ‘low-tech' to high-resolution structures.
